# An Interpretive Phenomenological Inquiry into Type 1 Diabetes and Work

**DOI:** 10.3390/ijerph22081200

**Published:** 2025-07-31

**Authors:** Emma Victoria Shiel, Steve Hemingway, Rajeeb Kumar Sah, Kim Burton

**Affiliations:** 1 Mental Health Research Group, Division of Nursing, Midwifery and Social Work, School of Health Sciences, Faculty of Biology, Medicine and Health, Manchester Academic Health Science Centre, The University of Manchester, Oxford Road, Manchester M13 9PL, UK; 2 The Department of Nursing and Midwifery, School of Human and Health Sciences, University of Huddersfield, Queensgate, Huddersfield HD1 3DH, UK; s.j.hemingway@hud.ac.uk (S.H.); kimburton1967@icloud.com (K.B.); 3 The Department of Allied Health Professions, Sport and Exercise, School of Human and Health Sciences, University of Huddersfield, Queensgate, Huddersfield HD1 3DH, UK; r.k.sah@hud.ac.uk

**Keywords:** type 1 diabetes, work, psychosocial impact, phenomenology, health, accommodations, occupational health, work ability, self-management

## Abstract

There is little qualitative research on the support needed by workers with type 1 diabetes to effectively self-manage at work and maintain work ability. In this UK study, 21 workers with type 1 diabetes participated in semi-structured interviews. The interviews were transcribed and analysed using interpretive phenomenological analysis and then characterised under the Psychosocial Flags Framework. Findings highlighted several obstacles to maintaining self-management, including systemic workplace issues (black flags), individual attitudes and beliefs (yellow flags), and workplace issues (blue flags). Participants generally lacked confidence in voicing their needs, emphasising a requirement for a more supportive, inclusive workplace culture. This indicates a need for employers to foster an environment where workers with T1D feel comfortable seeking support without penalty. Addressing unhelpful perceptions of T1D seems key to this, making increased knowledge and awareness crucial for the harmonious integration of T1D with work. But delivering effective interventions may be challenging, since they must account for the complex biopsychosocial interplay of obstacles to work ability that this qualitative investigation emphasises.

## 1. Introduction

Type 1 diabetes (T1D) is an incurable autoimmune disease where the body attacks insulin-producing beta (β) cells. This process causes the pancreas to stop producing insulin, which is essential for the body to regulate blood glucose. Due to a lack of insulin, people with T1D must administer their own, as well as engage in various means of self-management (including treating hypo/hyperglycaemic events, multiple daily testing and regulating blood glucose) [1]. Once the diagnosis is made, the major role of healthcare is education on self-management routines (as well as providing insulin and its delivery mechanisms). For people with T1D, effective self-management is key to survival and the prevention of complications [2]. But self-management can become difficult when other commitments (like work) require attention and are not adequately supported [3].

In the UK, the experiences of workers with T1D, and the support they can access, are shaped by both the National Health Service (NHS) and UK legislation. The NHS provides access to healthcare, which is free at the point of need. Legislation under the Equality Act requires employers to provide reasonable adjustments for workers with T1D who need them. However, there is little communication and understanding between healthcare and employers, resulting in workers with T1D still navigating stigma, disclosure dilemmas, and inconsistent employer support.

Work has been well-documented to take priority over self-management for people with diabetes, which poses serious health threats to people with T1D worldwide [3]. Therefore, understanding the experiences of workers in self-management is of interest. However, there is limited qualitative data on this topic [4], so the specific support needs of workers with T1D remain unclear. Managing a long-term health problem at work is a biopsychosocial challenge best explored through qualitative methods, which usefully can be underpinned by the Psychosocial Flags Framework [5]. This framework identifies and addresses psychosocial obstacles to recovery and work participation for workers with health conditions like T1D, using a system of coloured ‘flags’ to categorise the obstacles:

- Yellow Flags: Individual factors such as thoughts, feelings, and behaviours.
- Blue Flags: Workplace-related factors, including high job demands, low satisfaction, inadequate accommodations, or poor employer–employee communication.
- Black Flags: Contextual factors like financial strain, restrictive workplace policies, or social isolation.

Identifying relevant flags in respect of T1D at work will point to obstacles across the three psychosocial domains, solutions to which can facilitate strategies to support workers with workplace self-management, thus reducing the risk of progression into worklessness [5]. The aim of the study, then, is to better understand work and T1D by qualitatively exploring the obstacles to workplace self-management reported by workers with T1D.

## 2. Materials and Methods

A qualitative interpretive phenomenological approach was used to understand the lived experience of UK workers with T1D. This was carried out using the most recent methods of Interpretive Phenomenological Analysis (IPA) [6] and reported in accordance with the Consolidated criteria for Reporting Qualitative research (COREQ) guidelines [7] and also adheres to the four markers of quality by Nizza et al. [8]. Overall, the research followed an idiographic approach, which typically involves smaller sample sizes than quantitative research to give participants a clear voice while minimising workload [9,10].

Twenty-one working-age individuals diagnosed with T1D were recruited (see Table 1 for detailed demographics). Convenience sampling (through the use of social media, flyers, and word of mouth) was used to recruit participants with notable effort to gain variation on the sample regarding characteristics such as job roles, methods of managing their diabetes, and age. Recruitment and data collection occurred simultaneously between April and May 2024, until theoretical saturation was met and the interviewer felt confident that enough data was gathered on this topic. Ethical approval was obtained from the University of Huddersfield’s School of Human and Health Sciences, specifically from the School Research Ethics and Integrity Committee (SREIC) in October 2023 (Reference Number: SREIC/2023/055). No specific ethical concerns were identified given the study’s low-risk nature and voluntary, anonymised participation.

Semi-structured interviews facilitated in-depth qualitative exploration into the lived experiences of having T1D in work. Each participant was interviewed once, using Microsoft Teams, for an average of 60 min. While flexible in structure, each interview was guided by a schedule, developed from the previous literature reviews and collaboration with the academic team (SH and KB). Questions covered topics like self-management, containment, psychological impacts, prejudice, and workplace support. To gather comprehensive data, all questions were open-ended, encouraging storytelling and tangents, allowing participants to freely share thoughts they felt were meaningful in explaining their day-to-day experiences. Questions were adjusted as needed to fit each participant’s unique situation. A pilot interview with a person living with T1D was initially conducted to gain valuable feedback to inform adjustments and refine the interview process.

Naturally aligning with the theoretical underpinnings of this project, IPA was the chosen analytical method for understanding the lived experiences of people with T1D in the workplace. Accordingly, the following steps were taken: Reading (and re-reading), Making Exploratory Notes, Making Experiential Statements, Clustering, Move on to the next case, and Looking for patterns across the cases. Following completion of all steps, the findings were synthesised and reported narratively. Importantly, the analytical process continued within the write-up, helping to weave the themes together into a cohesive narrative.

No compensation was provided for participation, meaning deception is unlikely. Participants were not asked for specific proof of diagnosis. However, diabetes tended to be present at the time of interview and the interviewer (EVS), who also has T1D, felt confident that the depth to which each question was answered showed participants to be truthful about their health status. Following Smith’s [11] advice, respondent validity checks were not conducted because the interpretations might be too abstract for participants to validate without understanding the IPA process. Additionally, power imbalances could make it challenging for participants to disagree with researchers, leading them to provide more socially desirable answers [12].

## 3. Results

**Figure 1:**
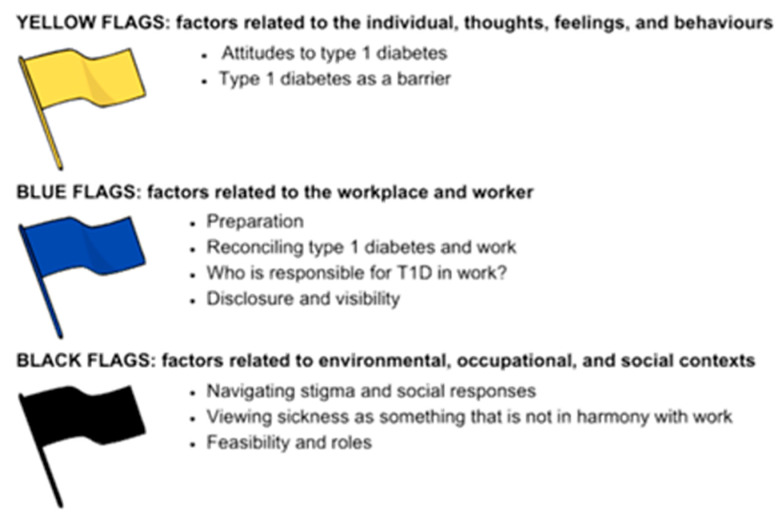
Themes in the context of the Psychosocial Flags Framework.

Workers with T1D face a complex and solitary workplace journey across three key areas. These findings are characterised under the Psychosocial Flags Framework. Using this framework can guide thinking, understanding, and action, providing a clear and systematic structure to analyse and address the complex interplay of personal, workplace, and contextual factors that influence participants’ lived experiences (see Figure 1).

To summarise:

Themes under ‘yellow’ flags represent psychological factors, focusing on participants’ thoughts, feelings, and behaviours. In this context, yellow flags tell the story of how it feels to live with T1D, and how that ‘permanent’ experience shapes participants’ meaning-making and influences their inner world. This theme also provides the basis for lived experiences and interpretations occurring throughout the rest of the analysis. Findings reveal that T1D requires continued daily management, creating pressure to maintain control and cope with negative self-perceptions. Many participants view T1D as a barrier to career success, adopting either “warrior” (open) or “victim” (concealing) mindsets that affect self-management.

Themes under ‘blue’ flags represent occupational factors, concentrating on the workplace and the relationship between work and health. Themes under blue flags centre around how participants feel about their T1D at work, and whether they are open about having the condition or prefer to conceal it. This theme also explores how participants go about prioritising their commitments, both to work and T1D, and how well that works for them. Findings show that balancing T1D with work responsibilities often leads to prioritising work over health due to fears of appearing less capable. Workers feel solely responsible for T1D management at work, while lack of awareness about disclosure processes and associated shame create significant barriers to seeking support. Distrust in workplace support reinforces isolation.

Finally, the themes under ‘black’ flags represent external and contextual factors that are beyond participants control, such as workplace policies and societal attitudes. Unlike the yellow and blue flags, themes falling under the black flags reflect the actuality of the lived experiences of people with T1D at work, exploring the aspects that are out of their hands, such as the requirements of the role or other people’s opinions of their T1D. Findings reveal widespread lack of T1D knowledge among colleagues, creating distress and misconceptions. Workers often feel that showing illness implies weakness, leading to avoiding time off and apologising for health needs. Some experiences suggest that some job roles are incompatible with T1D management, forcing compromised self-care. Additionally, many seem unaware of employment rights, saying that current legislation feels outdated.

Each theme is presented in further detail below.

### 3.1. Yellow Flags: Factors Related to the Individual, Thoughts, Feelings, and Behaviours

#### 3.1.1. Recognising Type 1 Diabetes as an Individualised Condition

Experiences of T1D were unique to each participant but felt solitary despite shared patterns. Participant 20 illustrates this:

“…it’s just such a complex unique condition to each person… if there was almost like a diabetic robot of sorts and you gave it to somebody else; it wouldn’t work for the next person alone. You couldn’t do a black and white job per person”.

This highlights T1D’s complexity and individuality, suggesting it cannot be managed uniformly. Participant 1 captures this by using their T1D journey to showcase strength and resilience, leveraging it for benefits but facing concerns about discrimination:

“I use my diagnosis story and how I treat my type one diabetes to show my strengths, and it definitely helped get my university grant. But it’s something that I’ve definitely struggled with, especially in that self-identification of disability and whether it’s a disservice to myself, and my career”.

Conversely, Participant 7 exemplifies a proactive approach:

“I wanted to join the police force when I was a kid, couldn’t join the police force because I was diabetic. But then found out I could join the British Transport Police. So, I looked at doing that. Diabetes will not define me. And if there’s a way that it’s stopping me, I will find a way around it.“

This reflects a mindset focused on adapting and overcoming obstacles, integrating T1D into a positive identity framework.

#### 3.1.2. The Reality of Type 1 Diabetes

Participant 19 reflects on the long journey to confidently managing T1D, sharing “it has taken a lot of years to get to this point”. This sentiment is echoed by Participant 3, who emphasises the importance of self-compassion when self-management efforts falter:

“Yes, it really is a lot to think about. And even socially, I mean, I love to walk and stuff like that. But walking initially proved a nightmare because I’d bolus, I’d have breakfast. I’d go out walking and I’d hit a low within half an hour. What the hell? So again, learning to do that and being kinder to myself”.

Her detailed description of managing T1D highlights the complexity and relentless nature of the condition. Participant 2 acknowledges this, noting that “everyone has to learn the hard way,” reinforcing the idea that managing T1D involves enduring unpredictable fluctuations and occasional self-blame, as noted by Participants 1 and 9.

Participant 21 adds insight into the frustration of managing T1D, especially when unexpected fluctuations occur:

“They’re [other PWD on social media] the unicorns of today and that’s not actually everybody every day. That’s very rare and coming to that realisation is helpful. It doesn’t mean I don’t get fixated on it every now and again. Especially when it just does something completely against what you know to be true. That’s when you’re like, I don’t understand how this has happened, and I start sifting back through data and checking what I took and what I was and all that sort of stuff”.

The pressure to maintain control is a common theme, as demonstrated by Participant 7, who comments, “if you’ve got to that state, then you’re obviously not very well in control,” highlighting the negative self-perception tied to T1D management.

Participant 21 explains how technology like Continuous Glucose Monitors can influence self-esteem:

“If you only got 85% TIR, you’re not good enough”.

Despite their intention to aid in managing blood glucose, these statistics can lead to self-scrutiny. Those with better averages generally felt more confident and freer from T1D’s constraints, as seen with Participant 7:

“If there’s a way that it’s [T1D] stopping me, I will find a way around it”.

Conversely, Participant 15 shares the debilitating impact of T1D:

“I mean T1D affects everything. It affects everything for me originally because it was so debilitating to me. It changed everything, I’ve lost so much from it”.

This persistent struggle with T1D links back to the complex nature of the condition, leading to varied perceptions of identity among participants as either a ‘victim’ or a ‘warrior’.

#### 3.1.3. Being a Victim or a Warrior

Several participants felt victimised by T1D. Participant 5 shared, “I was so different at school. When everyone started wearing tight outfits, I clipped my insulin pump to my bra. You could see it. I haven’t got past that trauma of being different with it, which sounds a bit ridiculous and especially when I know it would make everything so much easier for me”.

Participants with these negative feelings often concealed their health status, even outside the workplace. Participant 1 said, “I kind of went along with my urge to conceal as much as I could, even outside of work, my type one diabetes is something I don’t want to be seen or heard about”.

Conversely, those who saw themselves as ‘warriors’ were more open about their experiences. Participant 3 noted, “I’m not hiding in the corner. I want to be a warrior through T1D, rather than a victim”. Her words suggest a battle against societal pressures and a struggle to self-advocate confidently. But participants knowledge about workplace rights fluctuated.

Negative narratives about T1D often resemble ‘horror stories,’ leading to frustration due to the lack of positive examples. Participant 4 highlighted the perceived divide between T1D and T2D, saying, “If people don’t know the difference between type one diabetes, which is uncaused and unfortunate, compared to Type 2, where the stereotype is sort of fat”. This comment reflects a sense of othering and judgment within the diabetes community. Associations with T2D were particularly disliked, as many participants felt T2D was viewed as self-inflicted and less challenging. Participant 12 emphasised that managing T1D requires constant awareness and effort, contrasting with the perceived simplicity of T2D.

Participants’ interactions with other PWD determined whether T1D was a shared, supportive journey or a solitary one. Given the complexities of T1D, variations in treatment, technology, and attitudes can make it challenging to relate to others, as Participant 9 stated, “Ultimately, it’s always been me”.

#### 3.1.4. Perceiving Type 1 Diabetes as a Barrier or Motivator

Generally, participants felt pessimistic about their T1D, viewing it as a constant physical and emotional strain. Despite their efforts, T1D was seen as a barrier to success and an added emotional burden.

Participant 15 described how T1D exacerbated her struggles, causing her to leave work due to the compounded difficulties. She highlighted T1D as the tipping point of her breakdown, making it hard to balance other commitments. This sentiment was echoed by Participant 3, who described T1D as “like having a full-time job within itself”. Participants often compartmentalised T1D as a separate issue, requiring significant attention, as noted by Participant 10’s struggle to juggle “180 other things a day”.

The constant strain led to feelings of debilitation, especially in their work lives, forcing many to choose jobs that were compatible with T1D rather than their desired career paths. This limitation impacted their self-esteem, as they felt capable yet restricted. Participant 8 expressed this as a perpetual worry, feeling held back by T1D: “It feels like it holds me back a little bit because it’s the first line of everything”.

Such barriers prompted participants to reflect on how their lives might be without T1D, with some, like Participant 5, wishing to be free from diabetes, while others, like Participant 6, calmly accepted it as “just the reality of life”. The necessity of strict self-management for survival brought significant emotional burden and grief, as participants grappled with the impact of T1D on their ability to thrive.

### 3.2. Blue Flags: Factors Related to Perceptions of the Workplace and the Work-Health Relationship

#### 3.2.1. Preparing for Work

As T1D is already ‘all-encompassing’, when work responsibilities are added, conflicts can arise. Participants had different approaches to managing T1D at work, with some preparing extensively and others being more reactive. Lack of preparation can turn daily routines into unpredictable challenges. Participant 2 described how not having medication at work endangered her life:

“I had a bad low, didn’t have sweets, and didn’t ask anyone. I walked to the shop and thought I would die. Sitting in a sweet shop, embarrassed, all I could think was I’m going to die. This could have been stopped if my manager had provided a drawer or locker for medication”.

This highlights the extreme consequences of being unprepared. The fear and embarrassment of tarnishing their professional image were common, underscoring the importance of preparation. Some participants, despite knowing its importance, refused to prepare. For example, Participant 5 used workplace sugar sachets to manage hypoglycaemia discreetly, showing a disinterest in monitoring blood glucose at work while prioritising image. This reactive approach contrasts with others who “make sure ahead of time” (Participant 8).

Participant 7 stated:

“You have to be prepared. You’re going to have stressful periods”.

This emphasises the importance of planning and the inevitability of stress. Managing T1D at work requires significant effort and concentration. Participants viewed themselves as the sole managers of their T1D, highlighting the solitary nature of self-management.

Participant 12 described always carrying ‘carbs’ and a bag as a safety net, highlighting the burden of preparation. This constant need to carry necessary items suggests a mental and physical burden. Participant 5 expressed gratitude that “bags are now fashionable” to avoid standing out, indicating an ongoing sense of feeling different. Participants felt most comfortable when they had control over T1D at work. However, the unpredictable nature of T1D meant accepting a loss of control, causing panic.

Unpredictable work schedules caused significant anxiety. Not knowing when breaks would happen made managing blood glucose harder, adding stress:

“…they’re the days that will get us, and they’re the ones when things are outside my control. It could be a team meeting, and you don’t know when you’ll stop for lunch. I’m used to having lunch at 12:00, but they have it at 1:00. These things really throw me off” (Participant 17).

Participants often wondered about break times mid-meeting, which distracted them and affected productivity.

#### 3.2.2. Reconciling Type 1 Diabetes and Work

It is clear that managing T1D requires considerable personal effort: balancing it with professional responsibilities can be difficult and requires careful preparation, which makes it difficult for workers to perform at their best due to being preoccupied with their condition. This highlights the importance of a flexible and open work environment supportive of these needs. Participants found a structured routine helpful for maintaining control at work.

Dealing with a loss of control at work was difficult for participants, as many did not want to ‘make a big deal out of it’ due to concerns about appearing ‘less’ capable, particularly as they are already categorised as disabled. Participant 14 said they ‘did not even think about it’ when it came to self-management. To avoid this, some participants split their attention between T1D and work, discreetly managing their health to blend in while maintaining productivity. However, this often resulted in prioritising work over health.

Concerns about appearing distracted and the pressure to perform led participants to act ‘like a non-diabetic person’, prioritising work over their health. This pressure often came from the workplace, leading to presenteeism. Many participants believed that their role did not permit self-management and felt that disclosing their health struggles could lead to financial loss and negative consequences—especially those in casual roles.

Participant 9 explained:

“My boss came in, like the big boss, and she was like, how was that then? And I went. God, I’m knackered. Like I’m so excited to go home and, like, drink some water and sort my blood sugars out, I feel atrocious. And she was like, oh, right. Sorry, sorry about that. And then I got cancelled for the next 3 shifts and I lost a lot of money. And instead of earning like 500, 600 pounds for that month I earned like 200, which meant that I had to go into my savings to pay my rent”.

Participants generally wanted to stay healthy, but psychosocial obstacles often led to prioritising work, resulting in working while ill. Financial motivation reinforced this behaviour, as maintaining a strong work ethic brought financial security.

Interestingly, only two participants (who were in permanent, senior roles) diverged from this pattern. They advocated for themselves, putting their health first and feeling confident in their ability to manage T1D at work. It is possible that the nature of their roles provided the flexibility to prioritise their health, as they faced minimal risk of job insecurity or income reduction.

Participant 12 said “I’ll come when I’ve eaten,” and noted that managing T1D at work was no more difficult than in normal life. Participant 21 expressed concerns about hyper-fixation on perfect self-management impacting their job performance, stating “I get too fixated at work about it. Like I have the same breakfast, same everything. It’s completely disordered; I’m obsessed with my diabetes being perfect at work because I have to perform. But at the same time, I’m spending more time on my diabetes and fixating on that than getting my job done”.

Overall, the workers’ own thoughts tended to act as barriers to achieving harmony between T1D and work, rather than the workplace itself. While the workplace does present obstacles (see black flags), participants often mis-managed due to their own expectations—perhaps reinforced by workplace norms. Managing T1D and work often felt like a zero-sum game, where prioritising one aspect could lead to negative consequences in the other. Participant 13 stated, “work gets in the way and the diabetes gets in the way,” and Participant 4 explained how it is easier to focus on one at a time “rather than trying to delineate the two”.

#### 3.2.3. Who Is Responsible for T1D in Work?

The idea that responsibility dictates whether someone seeks support is a key theme. Most participants did not view their health as their employer’s responsibility, feeling solely responsible for their T1D. Thus, relevant legislations and protections were not exercised. Participants exhibited a strong sense of independence and confidence over their bodies, knowing they were the best managers of their condition. They generally took pride in their daily efforts to stay alive. For example, Participant 2 said

“There’s that real pride of like I’m my own person. I’m managing myself”.

This sentiment was echoed by Participant 3:

“I’m responsible for my body”.

Participants tended to manage their T1D without relying on external assistance, feeling empowered. However, this independence can lead to self-blame in cases of failure:

“My diabetes control just went really, really shit for no apparent reason. And I was working so hard...I’ve clearly done something wrong. I need to fix it” (Participant 13).

Participant 13′s words illustrate the detrimental effect of extreme self-sufficiency, leading to self-criticism despite constant efforts. Participant 15 shared a similar sentiment:

“When you get sent into that room with a Nurse saying...you have to inject now for the rest of your life...but it means I’m a pretty independent person and I just get on with my own shit...No point raising it, because what can happen?”.

He reveals a stoic attitude, enduring hardships silently. Some participants diverged from complete self-sufficiency, suggesting that workplace support could help:

“T1D is your responsibility, but you need support to maintain that drive” (Participant 8).

For Participant 8, managing T1D is his responsibility, but having support helps him stay motivated. He acknowledges the fatigue associated with T1D, suggesting that support can empower effective self-management.

#### 3.2.4. Disclosure and Visibility

Participants had little knowledge on where and how to disclose their health conditions at work. They seemed disconnected or uninformed about available support systems and their rights. Participant 9 stated she had ‘literally no idea,’ indicating a lack of support and guidance. This suggests she either chooses not to seek this information or feels she has no one to ask. Participant 21 admitted, “not sure how you even go about it,” appearing reluctant to seek the necessary information. This hesitation might stem from fear of potential repercussions, as Participants 10 and 20 recall being questioned about their ability to continue working after disclosing their T1D. This demonstrates how disclosure can alter perceptions of a person’s work ability. The associated risks were substantial and often outweighed the benefits, leading many participants to conceal or be deceptive about their condition. This presents living with T1D at work as primarily the individual’s responsibility, reinforcing a theme of solitude and self-reliance. Each challenge faced by participants builds towards a central message of being self-sufficient or alone. The portrayal of self-managing T1D in isolation ties together the different themes, underscoring the importance of resilience and inner strength.

Shame was a distinct barrier that stopped participants from accessing or engaging with support and schemes at work. Participants tended to discreetly manage emergent situations, often pretending to be well when they were unwell. To maintain this facade, many participants administered insulin or changed infusion sets in bathrooms. Participant 10 admitted doing this due to feeling ‘ashamed,’ fuelled by colleagues’ lack of understanding and empathy. She later states, “if they were to be in our shoes,” highlighting the differences between their realities and the T1D experience. Participant 7 confessed to injecting insulin through his jeans, attributing this to laziness and secrecy, suggesting viable rooms were inconvenient. When challenged about safety, he stated, ‘so the needle bends, you know, big deal,’ highlighting his nonchalance towards self-management choices. Similarly, Participant 8 discusses how he has become “quite adept at doing it without somebody realising,” suggesting pride in concealing his T1D. Like others, he enjoys protecting his work persona to avoid looking ‘sick.’ However, delays in administering insulin led to poor self-management and frequent illness. Participant 9 shares that

“…it does feel like a dirty secret a lot of time because you’re constantly playing it down and it’s like you’re always on, what’s the word… like damage control because you can’t be, like, clean about it for one sec and say actually, I’m dying. I’m really, really high and I really don’t feel well”.

This quote encapsulates how severe shame and illness interact. The more unwell she gets, the harder it becomes to admit. Like others, she consistently ‘plays it down,’ insinuating the truth would be detrimental at work. She must limit what others see or know to protect her work persona. This suggests she perceives T1D as not having a ‘place’ in work, implying it would be inappropriate or wrong to discuss. She talks about dying, showing glimpses into existential thoughts about T1D. Her feelings centralise around feeling ill, as she comes to terms with how sick she truly feels. Yet she withstands this, as she must keep her vulnerabilities hidden. Participant 17 felt the need to ‘hideaway and not tell anybody,’ revealing how shame controls their life. It becomes clear that many participants feel the risk of intense illness is worth it to avoid being honest about their health.

Trust within the workplace environment played a critical role in how participants approached T1D management. Few participants had faith that their T1D would be welcomed or supported by co-workers. Participant 15 recalled, “but instead of support to help with diabetes and mental health after about six months, I got taken into the office and told that I need to speed up my work”. Because of this, there was little value in disclosing. Participant 16 stated, “there was no point talking about my illness to them,” demonstrating a lack of payoff. She refers to her colleagues as ‘them,’ illustrating how she feels different or separate from the team, presenting the participant as alone. This resulted in tension as it created an ideological division. For some, this led to conflict, evident with Participant 14 who told their manager to ‘shove’ the job due to poor accommodations. Participant 1 recalled feeling anger from her supervisor, feeling ‘inconvenienced’ by her T1D. She ‘feels’ not hears his anger, suggesting his emotions are non-verbal. This frames interactions in work as negative, as colleagues without T1D are viewed as outsiders. These feelings add to the stigma and shame. Participant 1 later discusses feeling like “such a bad diabetic,” highlighting strong connections to feelings of judgement and alienation. Ironically, this belief leads her to perform poorly in both self-management and her job.

Interestingly, when T1D becomes visible, feelings of judgement increase. Visibility tends to be because participants are unwell, encouraging feelings of inadequacy. Thus, many participants feel it is better to conceal their T1D, protecting their image and emotional state. However, not every participant felt this way. Participant 12 discusses not being embarrassed about their T1D, yet also not wanting attention:

“I don’t know. It can be weird when people point out my equipment. But I don’t hide it, but if I need to fiddle with my pump, I’m not worried about people seeing it. But then again, I don’t show it off either. Not too much attention”.

This quote implies a balance between acceptance and cautiousness. They appear comfortable with T1D being visible yet prefer little attention. Their behaviour is nuanced, neither concealing nor flaunting it. This could be understood as trying to maintain normalcy without making T1D a focal point at work. This was also seen with Participant 4 who described not wanting T1D to be ‘the front and centre’ in working relationships. While not actively hiding their T1D, their experiences differ from Participants 13, 19, and 21 who describe themselves as ‘loud and proud’ about their diabetes. These participants feel there is ‘nothing to hide.’ They recall a journey of self-acceptance, becoming confident as a person with T1D and not letting it hold them back. Participant 19 was influenced by working with others with LTCs, including T1D, and by gaining higher authority and job status. This progression possibly made their job feel more secure, enabling them to reveal vulnerabilities. Ultimately, participants’ attitudes towards T1D and confidence to self-advocate were indicative of their experiences. However, the impact of co-workers, particularly superiors, must not be ignored. As Participant 2 stated, “If you have an unsupportive manager, your life is going to be just so difficult”. This highlights the significant impact that a manager’s support—or lack thereof—can have on PWD’s work life.

In conclusion, reconciling T1D and work is generally perceived as extremely challenging, if not impossible. Doing so effectively required extensive preparation, which some participants found easier than others. Participants agreed they must take responsibility for their T1D, but this often led to solitary experiences. While some acknowledged that work should play a supportive role, they ultimately view T1D as their duty to manage. Visibility plays a significant part in this dynamic, as participants wanted to present themselves as healthy and fit for work. This meant some participants went to great lengths to prove their workability, often omitting self-management and disclosing little about their condition. Although a few described themselves as ‘loud and proud,’ the majority struggled immensely, describing their experiences with T1D at work as difficult, frequently managing the tension between being good at T1D and their job simultaneously.

### 3.3. Black Flags: Factors Related to Environmental, Occupational, and Social Contexts

#### 3.3.1. Navigating Stigma and Social Responses

Participants were distressed at their colleagues’ lack of knowledge about T1D. Participant 11 mentioned a “blind spot” around understanding T1D, causing PWD to feel unsafe and misunderstood at work. Participant 1 emphasised that this lack of safety is normal, leading to a mindset of pushing through:

“I think fellow diabetics know that it’s not always safe at work. But when it’s your normal, you get into the, just push through it mindset. It just becomes your norm”.

The absence of knowledge about T1D was detrimental to participants’ comfort. Participant 9 feared that if she had a seizure at work, she might die due to the lack of colleagues’ awareness about emergency procedures. This sentiment was echoed by Participant 5, who wished people understood the severity of T1D. Similarly, Participant 9 noted that understanding the reality of T1D might scare people, implying widespread misconceptions.

Participant 12 shared that colleagues often panicked at the sound of her diabetes equipment, leading her to mute her alarms and experience erratic blood glucose levels. These “blind spots” could lead to uninformed opinions and negative impacts on self-management. Participant 2 and 19 mentioned that colleagues had “funny ideas” about T1D, which can lead to frustration and feelings of being misunderstood.

Participant 10 recounted an instance where a peer believed T1D was infectious, highlighting the extreme lack of understanding. This led to feelings of being othered or alienated. Participants 5 and 13 felt that colleagues’ actions created a sense of division, further contributing to isolation.

Participant 21 illustrated the lack of accommodations at workplace events, feeling excluded due to the absence of sugar-free options. This issue was not raised with colleagues, suggesting a pattern of silence due to previous negative experiences. Participant 10 shared that her manager had never helped, and Participant 6 avoided using accommodations in the presence of senior stakeholders to protect her reputation.

In conclusion, the social dynamics of work and T1D presented significant challenges. Many participants felt “othered” due to their condition and avoided seeking support to prevent negative interactions and blend in with their colleagues.

#### 3.3.2. Viewing Illness as Something That Is Not in Harmony with Work

Viewing illness as something not in harmony with work was common among participants. Many internalised the belief that showing signs of T1D could be seen as weakness or incompetence. For example, Participant 10 preferred calling her T1D a ‘condition’ rather than a ‘disease’ to avoid the negative connotations of chronic sickness.

Participant 3 rejected the term ‘recovery’ because it implies she is unwell. This highlights the idea that presenting as unwell could lead to different treatment or affect future job prospects. Being ‘well’ was seen as a hireable quality, while being ‘unwell’ was considered undesirable. Participant 5 felt embarrassment when tending to her T1D at work. She admitted, “I will begrudgingly fix it. And hate it.”

Participants felt the need to ‘toughen up’ and avoid taking time off work to prove they were well. Participant 4 worried that colleagues might presume he was slacking off if he took time off. Participant 20 found it difficult to disclose sickness, reflecting anxiety about workplace expectations and job security.

Participant 1 constantly apologised when managing her blood glucose at work, feeling guilty for focusing on her health rather than work. This behaviour shows how workplace social norms exert pressure on PWD to fit in, leading to poor self-management during work hours. If these harmful social norms persist, experiences of managing T1D at work will remain challenging.

#### 3.3.3. Feasibility and Roles

Away from social issues, another factor that influenced participants’ experience of balancing T1D and work was the job role itself. Participants worked in a variety of roles (see Table 1). Some complimented their self-management, while others were ill-suited. Voiced by Participant 2 “some work environments are not as forgiving for people with diabetes”, which can lead to mismanagement, and eventual complications. However, as participants roles were typically something they did not want to—or could not afford to—lose they tended to omit or alter their self-management to fit the role. This was instead of adapting their role to their T1D. Because of this, poor blood glucose management was common across participants in work hours.

“We have factory audits in the food industry where you’re tied up for hours, I would make sure my blood sugar was high before I went in” (Participant 7).

Intentional hyperglycaemia was common among participants to avoid tending to T1D during work hours; they viewed hypoglycaemia as more disabling than hyperglycaemia.

Manual jobs posed significant challenges due to physical demands. Participant 15, a labourer, shared how his T1D led to his dismissal due to complications and neuropathy issues. Despite the legislation offering protections for workers, negative social situations may lead to less engagement with their rights. Strength was fundamental to his job, but hypoglycaemia rendered him unable to perform his duties. He recalls, “this lad turns to me and says you’re not strong enough to do what you’re doing in this job. You’re not strong enough. Don’t come back.” Others like Participant 13, a general practitioner, prioritised patients over T1D management, and Participant 14 didn’t want T1D to be the reason for team failure.

Some participants found certain jobs incompatible with their needs. Participant 6 emphasised the dual responsibility of individuals and employers in managing T1D at work. Participants must choose jobs that complement their self-management instead of trying to fit into unsuitable roles. Participant 6 stated, “So, it’s definitely got to be a dual responsibility. But work definitely needs to support it in whatever way is realistic and feasible.” Those who actively managed their health maintained employment and higher self-esteem. Conversely, mismanagement led to complications and job loss, as seen with Participant 20, who lost his sight and job due to poor health management. He explained, “I didn’t lose my sight and become blind as such, that my vision became worse and worse until I needed intense help. And at that stage I couldn’t see any words, numbers, anything, any detail on a laptop. So, I informed my employer, who said we’ll have to let you go.”

While it may be challenging for PWD to accept setbacks, doing so is essential for their health and job security. These findings indicate that participants who actively manage their health are more likely to maintain employment due to being able to carry out their roles consistently and with fewer complications. Equally, being in a job potentially contributes to higher self-esteem, as participants in stable employment felt they were able to psychologically overcome their diagnosis. The opposite was observed in Participant 15, who experienced poor self-esteem from job loss and poor workability.

In conclusion, finding a balance between overcoming setbacks and accepting limitations leads to better personal and professional outcomes for PWD. They must carefully consider job compatibility with their T1D, as some roles inherently have limited flexibility. As Participant 7 advised, “Knowing your body, knowing what you can do, and knowing what works and what doesn’t” is crucial for achieving a balance and reconciling T1D and work.

#### 3.3.4. Employment Rights and Accommodations

While choosing the right job was crucial for participants, knowing and applying their employment rights offers additional support for self-management at work. Many participants were unaware of their rights or did not find them useful. Participant 17 expressed, “I don’t really know my rights. I haven’t really felt the need to go looking for them”.

Participant 20 felt that current rights did not help those with T1D, suggesting that legislation must be updated to reflect modern realities for people with T1D. Participant 18 noted, “technology has come an awful long way in the last 10 years...some processes and policies are a little bit dated”.

Participant 19 emphasised the need for changes in workplace culture over tangible modifications, stating, “they don’t have to necessarily be elaborate adjustments”. Participant 1 highlighted the importance of attitude change, saying, “There needs to be big changes in attitudes before anything”.

Acknowledgment and proactive offers of help were seen as empowering. Participant 2 shared, “Someone offering rather than you asking is just so much more powerful”. This sentiment was echoed by Participant 15, who appreciated when workplaces showed awareness of their T1D struggles.

Participants sought more tolerance and flexibility regarding time off and managing their condition. Better support and understanding were seen as crucial for effective self-management and workplace inclusion. Participant 13 concluded, “dialogue is where everything starts,” suggesting that open communication could drive improvements in work environments, including more helpful legislations and schemes.

## 4. Discussion

In summary, workers with T1D generally lack confidence in voicing their needs, highlighting the critical need for employers to create more supportive, inclusive cultures with enhanced T1D awareness.

The findings indicate that the lived experience of T1D is highly solitary and individualised. Participants viewed themselves as their own experts, but were concerned their illness would impede their career path—consolidating findings by Hakkarainen and colleagues [13]. Temporal aspects were common. Like Hansen et al. [14], participants constantly worried about their future. But those with positive ‘lifetime templates’ tended to feel less anxious [15,16].

Participants displayed a notable dichotomy in their approach to T1D—generally falling into either “warrior” or “victim” mindsets. Some participants were more fluid, rather than binary, sometimes being warriors and other times victims, highlighting how T1D can elicit a wide range of responses. This is similar to findings from Symonds et al. [17] who categorised workers with back pain as ‘copers’ or ‘avoiders’. Participants with a “warrior” mindset proudly incorporated T1D as part of their work persona and generally coped better at work. They showed greater confidence in advocating for their needs and overcoming challenges, meaning fewer absences. While those with “victim” mindsets tended to conceal their health. This often led to poor self-management which negatively affected health and workability—and potentially job loss [18].

This resonates with Hansen et al.’s [14] theory of ‘containment’ where workers with T1D modified their self-management to suit both work and T1D obligations. Aspects of the Social Cognitive Theory [19] could provide context to these differences—particularly the idea of self-efficacy in understanding the ‘victim’ and ‘warrior’ identity.

Participants reported a substantial lack of awareness around T1D, often leading to feelings of unsafety. This aligns with Wijk et al. [20] who found that PWD often feel distressed by others’ lack of knowledge, particularly in emergencies. A recent systematic review [21] analysed self-management interventions in UK workplaces, but none addressed raising general awareness or engaging colleagues, which the present findings suggest could be useful.

Consolidating the findings of Balfe et al. [22], that people with T1D may feel self-conscious about their condition at work, participants frequently expressed reluctance towards disclosure, feeling it positioned them as weak in the workplace. Many prioritised perceived workability over actual workability, hiding their illness to protect their professional image. Feeling awkward was common, negatively influencing help-seeking behaviours, and reinforcing self-reliance—a finding supported by the wider literature [23,24,25].

Many participants articulated the idea from Hansen et al. [14] of competing logics, which appeared distressing—a common feeling for people with T1D [22]. To reconcile this, several participants engaged in risky behaviours reinforced by previous ‘successful’ attempts where they avoided serious illness. Intentional hyperglycaemia and reactive self-management were key components to this which were also reported in the wider literature [26,27]. Broader data suggests T1D is often viewed as a personal issue separate from the workplace [28,29] leading participants to delay self-management until after work meetings.

That said, some participants successfully integrated both logics through consistent colleague support. Thus, supportive work environments could be a potential solution [30]. Colleague support has been widely accepted as essential for effective workplace self-management [25,29,31,32], supporting PWD to make health-promoting decisions and minimise tension [14].

Though presenteeism is traditionally viewed as harmful [33,34], the present findings suggest it may have positive implications, such as enabling continued employment and preventing potential downward spirals into worklessness by keeping workers with T1D in routines.

Finally, participants reported outdated workplace policies—a well-documented observation [24,29,31,35]. Participants stated that new policies must be flexible—something that has proven effective in supporting workers with complex needs [36]. But to enforce successful policies and interventions, continued collaboration between stakeholders and PWD must be considered to ensure mutually beneficial, and feasible, outcomes [21].

## 5. Conclusions

While this qualitative study allows for context-specific recommendations, the findings are not intended to be generalised within the UK or transferrable internationally. Nonetheless, they contribute to a growing body of evidence on the workplace experiences of workers with T1D. Over time, such cumulative insights may strengthen the basis for more definitive conclusions and policy guidance. The analysis reveals systemic issues related to workplace policies, stigma, and lack of awareness (black flags); there is a clear need for multi-faceted interventions that address both individual beliefs and attitudes (yellow flags) and workplace obstacles (blue flags). There is a general lack of confidence among participants in voicing their needs, suggesting a need to foster a more supportive and inclusive workplace culture. Employers may want to focus efforts on establishing a safer environment where workers with T1D feel they can speak about their health without being penalised, helping them to seek the support they need to be both productive and healthy in work time. In conclusion, addressing the contextual black flags, specifically how others perceive and act towards T1D, is crucial to ensure that workers with T1D can successfully remain in work. That said, interventions must simultaneously account for the biopsychosocial interplay between the different obstacles to work ability: the way that local workplace culture and practices (blue flags) shape individual attitudes and lived experiences (yellow flags) which, in turn, influence the effectiveness of attempts to overcome generic obstacles (black flags). Future research may focus specifically on strengthening the qualitative evidence base, which has recently been identified as both relatively sparse and rich with potential [37,38].

## Figures and Tables

**Table 1:** List of Participants.

No.	Ethnicity	Sex	Age (Years)	Age at Diagnosis (Years)	Diabetes Equipment	Role	Type of Employment	Sector	Size of Employer	Working Situation	How Important Is Self-Management? (1–10)	How Confident Are You to Self-Manage in Work? (1–10)
1	White Other	F	31	12	Omnipod 5 and Dexcom G6 [Closed-Loop].	Operations Manager.	Full Time [9 to 5PM].	Science and Research.	Small [Start-up Nonprofit].	Hybrid [Must be in Office for Tours].	10	5
2	White British	F	33	13	Dexcom G6 and Omnipod 5 [Closed-Loop].	Employment Specialist, Helping People with Mental Illness Return to Work (Band 5)	Full Time [9 to 5PM].	Health and Social Care.	Large [NHS].	Hybrid [Must be in Office for Patient Facing Activities].	10	10
3	African Indian	F	61	60	Dexcom 1 with Receiver and MDI.	Mix of Self-Employed Roles: Chartered Project, Professional, Board of Trustees, Trusteeship.	Varied [Most often 9 to 5PM but can work 12 h shifts].	Pan-sector.	Small [Self-owned, Participant is the only Worker].	Hybrid [Remote and International Travel].	10	8
4	White British	M	28	24	Freestyle Libre and MDI.	Analyst.	Full Time [9 to 5PM].	Services.	Large.	Hybrid [4 Days in Office, 1 at Home].	10	7
5	White British	F	29	11	MDI and sometimes wears the Libre.	Senior Purchasing Manager (One Level Down from Director).	Full Time [Hours Vary Depending on Workload].	Hospitality and Purchasing.	Very Large.	Hybrid [Works across Head Offices and Sites].	10	2
6	White British	F	33	29	Dexcom G6 and MDI.	HR Director, Talent Development.	Full Time [9 to 5PM].	Hospitality.	Large.	Hybrid [Flexible Working].	8	9
7	White British	M	54	4	Libre 2 and MDI.	Senior Manager.	Full Time [9 to 5PM].	Food Manufacturing.	Very Large.	Hybrid [Between Factory and Office].	10	10
8	White British	M	50	40	Tandem T-Slim and Dexcom G6 [Closed-Loop].	Senior Third Line Technical Support Engineer.	Full Time [9 to 5PM].	Information Technology.	Large.	Office Based.	9	7
9	White British	Non-binary	36	20	MDI and Dexcom 1.	Junior Public Engagement Assistant, University Archive Services.	Part Time 0.8 Hours.	Higher Education: Arts and Heritage	Large: but small team	Hybrid [Office 3 Days and 1 WFH].	8	7
10	White British	M	52	17	MDI and Libre 2.	Lower Management: US Law Firm [UK-based].	Full Time [9 to 5PM].	Law.	Large	Remote.	9	9
11	Scottish	F	59	9	Tandem T-Slim X2, and Dexcom G6 [Closed-Loop].	Structural Engineering Consultancy Communications Officer [Left due to T1D related illness that management would not accommodate].	Full Time [Condensed Hours].	Consultancy.	Small	Office [Occasionally Hybrid].	9	10
12	Eastern European	F	25	5	Tandem T-Slim and Dexcom G6 [Closed-Loop].	Event Builder [Manual Labour].	Ad Hoc	Entertainment/Event Planning.	Large	Site Based.	1	3
13	White British	M	56	51	Libre and MDI.	Machine Operative.	Full Time [9 to 5PM].	Industrial.	Large	Factory Based.	10	10
14	British Asian	F	56	25	MDI and Dexcom G7.	Self-employed Qualified Accountant [Retired due to ill health].	Full Time [9 to 5PM].	Finance.	Large [with experience in larger entities/offices].	Office Based.	10	10
15	Southern Irish	F	45	18	Omnipod Dash, and Libre 2 [‘DIY’ Closed-Loop, iPhone-based algorithm].	Database Officer.	Full Time [9 to 5PM].	Charity: Third Sector.	Large.	Remote Work with Travel.	8	4
16	White British	F	45	28	Medtronic 780 and Guardian [Closed-Loop].	Senior Doctor of Medicine [in Practice and Lecturing in Medical School].	Full Time [9 to 5PM].	Education and Healthcare.	Clinic: Small. University: Large.	Working Between Clinic and University.	8	6
17	White British	M	38	36	MDI and Libre 2.	Credit Specialist.	Full Time [9 to 5PM].	Finance.	Large.	Remote.	10	10
18	White British	M	32	7	Libre 2, and Omnipod Dash [Sometimes uses ‘DIY’ Closed-Loop].	Offshore Wind Turbine Engineer.	Full Time [9 to 5PM].	Engineering.	Medium.	Office and Offshore.	10	10
19	Black British	M	38	10	Omnipod Dash and Libre 3.	Senior HR Manager.	Full Time [9 to 5PM].	Human Resources.	Medium.	Hybrid [2 in Office, 3 Days at Home].	8	10
20	White British	M	47	8	Tandem-T-Slim and Dexcom G7.	Self-employed Online Business Owner [Selling Cosmetics and Beauty Items].	Full Time [9 to 5PM].	Consultancy/Information Technology.	Small.	Remote/.	10	9
21	White British	M	32	22	MDI, and Freestyle Libre 2.	Research Fellow [Early Career Researcher].	Full Time [9 to 5PM].	Research: Advanced Manufacturing and Engineering.	Medium.	Office Based [Occasional Remote Working].	9	6

## Data Availability

The datasets presented in this article are not readily available because of privacy or ethical restrictions.

## Abbreviations

The following abbreviations are used in this manuscript:

T1D	Type 1 Diabetes
HR	Human Resources
MDI	Multiple Daily Injections
IPA	Interpretive Phenomenological Analysis
PWD	People With Diabetes
